# Compassionate Love for a Romantic Partner Across the Adult Life Span

**DOI:** 10.5964/ejop.v13i4.1204

**Published:** 2017-11-30

**Authors:** Félix Neto, Daniela C. Wilks

**Affiliations:** a Faculty of Psychology and Educational Sciences, Universidade do Porto, Porto, Portugal; b School of Business and Social Sciences, Universidade Europeia, Lisbon, Portugal; Webster University Geneva, Geneva, Switzerland; The Maria Grzegorzewska University, Warsaw, Poland

**Keywords:** aging, compassionate love, love styles, subjective well-being

## Abstract

Compassionate love has received research attention over the last decade, but it is as yet unclear how it is experienced over a lifetime. The purpose of this study was to investigate compassionate love for a romantic partner throughout the adult life span, exploring individual differences in the propensity to experience compassionate love in regard to age, gender, religion, love status, love styles, and subjective well-being. The results showed that religion and love status display significant effects on compassionate love. Believers experienced greater compassionate love than nonbelievers, and individuals in love presented greater compassionate love than those who were not in love. Love styles and subjective well-being were found to be related to compassionate love. These findings corroborate studies that indicate that individuals who experience higher compassionate love for a romantic partner are more likely to report Eros, Agape, and subjective well-being.

Interest in understanding love has been growing since the mid-twentieth century, but much remains to be understood. In a recent contribution to the scientific study of love, Berscheid (2010) lays out a quadrumvirate model in which compassionate love (CL) is one of the four types of love experienced by romantic partners, along with passionate love, companionate love, and attachment love. She posits that, although these types of love can co-occur in romantic relationships, they are distinct from each other. Considerable research has been directed at understanding passionate love, companionate love, and attachment love, however, CL is still poorly understood (e.g., Berscheid, 2010; Fehr & Sprecher, 2009; Neto & Menezes, 2014; Oman, 2011).

CL is “the kind of love that ultimately centers on the good of the other” (Underwood, 2009, p. 3) and, although it is important to understand the course of CL across the lifespan (Fehr, Harasymchuk, & Sprecher, 2014), this area has not yet been investigated. The current study responds to calls for further investigation of CL (e.g., Berscheid, 2010; Fehr et al., 2014), aiming at extending research on CL for a romantic partner throughout the adult life span, and exploring individual differences in propensity to experience CL. With this purpose, an empirical study was conducted in Portugal focusing on the relationship between CL for a romantic partner and age, gender, religion, love status, love styles, and subjective well-being through time.

## Compassionate Love

Most definitions of CL contain the theme of giving of oneself for the goal of another (Shacham-Dupont, 2003). For example, CL was defined by Underwood (2009, p. 4) as “attitudes and actions related to giving of self for the good of the other”. Along the same lines, Sprecher and Fehr (2005, p. 630), defined CL as an “attitude toward other(s), either close others or strangers or all of humanity; containing feelings, conditions, and behaviors that are focused on caring, concern, tenderness, and an orientation toward supporting, helping, and understanding the other(s), particularly when the other(s) is (are) perceived to be suffering or in need”. CL can be experienced for a romantic partner, family, friends, peripheral ties, and humanity (Sprecher & Fehr, 2005). More recently, Fehr, Harasymchuk, and Sprecher (2014) reviewed the extant conceptualizations of CL and concluded that a common aspect is that they involve “extending beneficence to another” (Fehr et al., 2014, p. 580).

## The Current Study

The current research analyzed the CL for a romantic partner throughout the adult life span, and explored individual differences in propensity to experience CL. More specifically, the objectives of the current investigation were three-fold, as follows:

### Objective 1

The first objective was to explore whether there were differences in CL according to age, gender, religion, and love status.

#### Age

To date, there is relatively little investigation on the relationships between aging and the experience of CL. This is a serious lacuna. Researching the experience of older adults of CL is particularly relevant given the increase in life expectancy (Hatfield & Rapson, 2014). Past research on the relationship between age and CL is mixed. Smith (2009) found that empathy and altruistic values were most common in mid-life. However, this was not found for feelings of normative obligation to help kinfolk (Marks & Song, 2009) and altruistic behaviors (Smith, 2009), which were quite high among older adults. Given these mixed findings, we are not in any position to advance a specific hypothesis regarding the influence of age on CL, but we will address the following research question: How is age related to CL?

#### Gender

Past investigation has found gender differences in CL among college students (Neto, 2012b; Sprecher & Fehr, 2005), where women experienced CL for others to a greater degree than men, regardless of the target of CL. Hence, in this study, women were expected to experience more CL than men.

#### Religion

CL is a key element of religion and spirituality (see e.g., Oman, 2011). In her comprehensive model of CL, Underwood (2002) posited that the religious socialization of an individual affects the likelihood of being compassionate. Furthermore, research findings displayed an association between religiosity and the experience of CL (Sprecher & Fehr, 2005). Portuguese society has been dominated by Catholic tradition hence it was expected that believers would experience more CL than nonbelievers.

#### Love status

Falling in love is difficult to define. It is a selective orientation towards a particular individual (Hendrick & Hendrick, 1988) that can mirror hormonal changes and physical attraction, and “can actually lead to giving of self for the good of the other” (Underwood, 2009, p. 5). Given that someone in love is dominated by feelings of caring for one specific person, CL may increase with being in love. Thus, in this study, it was expected that individuals in love would experience higher CL for a romantic partner than those not in love.

### Objective 2

The second objective of this study was to examine the relationship between CL and other psychological constructs, namely love styles and subjective well-being.

#### Love styles

Lee (1973) put forward a comprehensive six-style model of love, with three primary styles and three secondary styles, by analogy with chemical compounds. The primary styles include Eros (passionate, romantic love), Ludus (game-playing love), and Storge (friendship love). Compounds of two of each of the primary styles form the three secondary styles: Pragma (rational, shopping-list love, a compound of Storge and Ludus), Mania (possessive, dependent love, a compound of Eros and Ludus), and Agape (all-giving, selfless love, a compound of Eros and Storge) (Neto & Pinto, 2015).

All the above love styles are different ways of loving as there is not just one way of loving (Hendrick & Hendrick, 1986). The Love Attitudes Scale is based on Lee’s typology and measures attitudes towards love. It was tested cross-culturally (Neto, 1994; Neto et al., 2000). Research with college students showed that CL is significantly and positively associated with erotic and agapic orientations, negatively associated with ludic and pragmatic orientations, and without association with storgic and manic orientations (Neto, 2012b).

#### Subjective well-being

Subjective well-being (SWB) focuses on personal evaluations of one’s life experience and has three components: satisfaction with life, positive affect, and negative affect (Diener, 2000; Updegraff & Suh, 2007). Satisfaction with life is a cognitive evaluation of one’s life satisfaction either globally or with respect to specific life domains based on criteria chosen by each person (Pavot & Diener, 2008). In the present work we considered global satisfaction and two specific life domains, namely satisfaction with love life and satisfaction with sex life.

Positive affect concerns occurrences of positive emotions such as joy, contentment, and happiness. Negative affect concerns the experience of negative emotions such as shame, sadness, and anxiety. Although cognitive and affective components of SWB are correlated, they form distinct factors (Lucas, Diener, & Suh, 1996) and thus, a comprehensive evaluation of SWB requires measuring both components. Past research reveals that individuals who experience CL for other partners are happier in their relationships. For example, Diener and Lucas (2000) found love to be a prominent predictor of SWB (e.g., Diener & Lucas, 2000), and Smith (2009) showed that altruistic love was related to happiness (Smith, 2009). Other studies have documented that satisfaction with love life is linked to happiness and satisfaction with sex life (Apt, Hurlbert, Pierce, & White, 1996; Neto, 2012a).

Based on the above, it was expected that satisfaction with life, satisfaction with love life, satisfaction with sex life and positive affect would be positively correlated with CL; and negative affect would be negatively correlated with CL across the adult life span.

### Objective 3

#### Predictors of CL

The third objective was to explore the best predictors of the CL for a romantic partner. Therefore, the following question was addressed: What are the best predictors of CL for a romantic partner? In order to answer this question, multiple regression models would be used to examine the relative strength of background variables, love styles, and subjective well-being in predicting CL. Significant predictors of CL were expected within each of the three sets of variables.

## Method

### Participants

All participants were dating someone or had a partner (were in a relationship with at least one month). The sample comprised 614 participants, 49% women and 51% men. The ages ranged from 26 to 90 with a mean age of 44.70 years (*SD* = 15.80). The mean age of the participants by gender was not significantly different, *F*(1, 613) = 0.001, *p* > .05. The participants were classified into three age groups in accordance with Erikson (1963)’s psychosocial stages: young adults (26-30 years old, *N* = 149), adults (31-59 years old, *N* = 315) and the elderly (60-90 years old, *N* = 150). Concerning religion, 73% of the participants reported being believers and 27% non-believers. Regarding the level of education, 21 percent had not concluded secondary education, 29 percent had completed the secondary level, and 50 percent had attended university. As for the marital status, 35 percent reported being single, 42 percent married, and 23 percent were separated, divorced or widowed.

### Assessment Instruments

The participants were assessed using six scales, previously adapted to the Portuguese population, in addition to questions pertaining to age, gender, religion, and aspects of relationships. All scales were scored in such a way that higher numerical values corresponded to higher levels of the construct being measured.

- *Love Attitudes Scale* – The 42-item Love Attitudes Scale (Hendrick & Hendrick, 1986) was utilized. Participants assessed their degree of agreement with the statements using a 5-point Likert scale. The Portuguese adaptation of this scale was previously performed by (Neto, 1994; Neto & Pinto, 2015). On this sample, Cronbach standardized alphas were .79 for Eros, .72 for Ludus, .78 for Storge, .83 for Pragma, .81 for Mania, and .79 for Agape.
- *Satisfaction With Life Scale* – This scale, originally developed by Diener et al. (1985) and adapted to the Portuguese population by Neto (1993), was applied. It consists of five items, such as: “I am satisfied with my life.” Respondents rated their degree of agreement with the statements using a 7-point Likert scale (from 1 = *strongly disagree* to 7 = *strongly agree*). This scale showed reliability and validity in the Portuguese population (Neto, 1993; Muñoz Sastre et al., 2003). On this sample, the Cronbach standardized alpha was .82.
- *Positive and negative affect* were assessed through the Positive and Negative Affect Schedule (Watson et al., 1988) and adapted to the Portuguese population by Simões (1993). This is a measure of positive and negative affectivity that includes 22 emotion adjectives. Respondents are asked to use a 5-point scale to indicate how often they generally experience each emotion. On this sample, the Cronbach standardized alphas were .81 and .90, respectively.
- The *Satisfaction with Love Life Scale* was previously developed by Neto (2005). This a five-item scale and an example item is: “The conditions of my love life are excellent.” Respondents were asked to rate their degree of agreement with the statements using a 7-point Likert scale (from 1 = *strongly disagree* to 7 = *strongly agree*). The reliability and the validity of this scale have been previously demonstrated (Neto, 2005). On this sample, the Cronbach standardized alpha was .90.
- *Satisfaction with Sex Life Scale* was previously developed and its reliability and validity demonstrated (Neto, 2012a). This scale includes five items, such as “So far I have got the important things I want in sex life.” Respondents were asked to rate their degree of agreement with the statements using a 7-point Likert scale (from 1 = *strongly disagree* to 7 = *strongly agree*). On this sample, the Cronbach standardized alpha was .88.
- *Compassionate love for partner* (Sprecher & Fehr, 2005). The 21-item relationship – specific version of the Compassionate Love Scale (CLS) comprises items such as, “One of the activities that provides me with the most meaning to my life is helping ___ [the partner].” Respondents rated their degree of agreement with the statements using a 7-point Likert scale (from 1 = *not at all true* to 7 = *very true*). The Portuguese adaptation of this scale was previously performed by Neto (2012b). On this sample, Cronbach standardized alpha was .89.

### Procedure

Data was collected by psychology researchers and respondents were contacted in a range of venues, such as, universities, shopping centers, and community groups. The response rate was high (71%). The questionnaire was completed in less than 30 minutes. The survey was conducted in accordance with the legal and ethical norms in the country and all participants were unpaid volunteers.

## Results

### Background Variables and Compassionate Love for a Romantic Partner

The mean score for the Compassionate Love Scale was 5.16, with a standard deviation of .89. Mean values for Compassionate Love Scale were calculated separately for each gender and for each age group. As previously said, participants were classified into three age groups (Table 1). The young adults ranged in age from 26 to 30, adults from 31 to 59, and elderly from 60 to 90 years old. The data were analyzed by means of a 2 x 3, Gender x Age group design. ANOVA results showed non-significant effects for gender [*F*(1, 614) = 0.87, *p* > .05] and age differences, [*F*(2, 614) = 1.15, *p* > .05]. The interaction gender x age was also not significant, *F*(2, 614) = 1.27, *p* > .05.

**Table 1:** Descriptive Statistics for Compassionate Love as a Function of Selected Background Variables

Variable	*M*	*SD*
Sex, *F*(1, 614) = 0.87		
Women	5.18	.87
Men	5.15	.89
Age, *F*(2, 614) = 1.15		
26-30 years	5.22	.92
31-59 years	5.18	.84
60-90 years	5.08	.91
Religion, *F*(1, 607) = 4.26*		
Believers	5.21	.85
Non-believers	5.04	.93
In love now, *F*(1, 613) = 16.22***		
Yes	5.21	.85
No	4.76	.97

*p < .05. ***p < .001.

The effect of religion on CL was significant, *F*(1, 607) = 4.26, η^2^ = .007, *p* < .05. Participants who reported being believers experienced more CL (*M* = 5.21, *SD* = .85) than those who were not believers (*M* = 5.04, *SD* = .93). There was also a significant effect of love status [*F(*1, 613) = 16.22, η^2^ = .026, *p* < .001]. Participants who reported to be in love experienced greater CL (*n* = 547, *M* = 5.21, *SD* = .85) than those who were not love (*n* = 66, *M* = 4.76, *SD* = .97).

The greater the mean, the greater was the CL score. Within each column, for each variable, means with no superscripts in common differed at the 0.05 level, either by *F* test directly for a pair of means or by Scheffe test for three or more means.

### Correlations With Other Variables

It was expected that the CLS would correlate with other psychological measures in predictable ways (Table 2). In the three age groups CL scores correlated significantly with Eros, Ludus, Pragma, and Mania. CL was positively and significantly correlated with Eros and Agape, and negatively with Ludus and Pragma. The only exception to this pattern was the non-significant correlation between CL and Pragma among older adults.

**Table 2:** Correlations Between Compassionate Love Scale Scores and Other Variables

Variable	Compassionate Love Scale			
	Young adults (*n* = 149)	Middle-age adults (*n* = 315)	Elderly (*n* = 150)	Total (*n* = 614)
Eros (romantic)	.39***	.22***	.30***	.28***
Ludus (game playing)	-.17*	-.11*	-.21***	-.14**
Storge (friendship)	-.02	.07	.14	.07
Pragma (practical)	-.22***	-.11*	-.02	-.12**
Mania (possessive)	-.14	.02	-.02	-.03
Agape (altruistic)	.44***	.25***	.37***	.33***
Satisfaction with life	.18*	.32***	.27***	.29***
Satisfaction with love life	.41***	.18**	.28**	.26***
Satisfaction with sex life	.36***	.27***	.14	.26***
Positive affect	.17*	.42***	.46***	.35***
Negative affect	-.16*	-.23***	.23**	-.21***

*p < .05. **p < .01. ***p < .001.

As predicted, all three satisfaction scores were significantly associated with CL scores, except satisfaction with sex life among older adults. CL was also positively and significantly correlated with positive affect and negatively with negative affect across the three age groups.

As the pattern of correlations in the three age groups was quite similar, multiple regression models were employed to identify the relative strength of the variables in predicting CL for the whole sample. On the first step socio-demographic variables (gender, age, religion, and love status) were entered into the model. On the second step the love styles were entered, and on the third step the well-being variables were added.

As shown in Table 3, the first set of socio-demographic variables predicted only 3 per cent of the variance in CL scores.

By adding the love styles variables, Agape, Pragma, Eros and Ludus emerged as significant predictors of CL scores. In addition, being in love remained a significant predictor. These variables predicted 21 per cent of the variance in CL scores. When the well-being variables were included in the final model, positive affect, negative affect, and satisfaction with life emerged as significant predictors. Moreover, Agape, Pragma, being in love and Eros remained significant predictors. Overall, the variables predicted 35 percent of the variance in CL scores.

**Table 3:** Multiple Regression Analyses of Background, Love Styles, and Subjective Well-Being Predictors on Compassionate Love for a Romantic Partner

Variable	*R*	*R^2^*	Beta	*t*
Step 1 Socio-demographic predictors (Stepwise)				
1. In love now	.16	.03	-.16	-4.03***
Step 2 Socio-demographic and love styles predictors (Stepwise)				
1. Agape	.32	.10	.32	7.77***
2. Pragma	.41	.17	-.27	-6.52***
3. Eros	.44	.19	.16	3.87***
4. Ludus	.45	.20	.13	-2.84**
5. In love now	.46	.21	-.08	-2.09*
Step 3 Socio-demographic, love styles nd well-being predictors (Stepwise)				
1. Positive affect	.35	.12	.35	7.99***
2. Agape	.47	.22	.32	7.89***
3. Negative affect	.54	.29	-.25	-6.37***
4. Satisfaction with life	.57	.31	.17	4.07***
5. Pragma	.58	.33	-.14	-3.25**
6. In love now	.59	.34	-.12	-3.08**
7. Eros	.60	.35	.09	2.10*

*Note.* The *beta* and *t* values are for the step at which the variables entered.

*p < 0.05. **p < .01. ***p < .001.

## Discussion

This work extends the investigation on CL to the entire adult life span. The CLS was presented as regards to a specific close other, most often a dating or marital partner. There are individual features that favor the expression of CL in individuals (Underwood, 2009), and in this study individual differences were explored in relation to the propensity to experience CL by focusing on age, gender, religion, love status, love styles, and subjective well-being.

In what concerns age variations in CL, the research reported here shows similarities over the adult life span. This result indicates that, rather than being a phenomenon largely confined to a specific age group, CL encompasses the entire life span. Hence, CL for a romantic partner is similar through life, which suggests that this construct, rather than being specific to a certain age, may represent a more universal standard.

No gender differences were found among people ranging from 26 to 90 years old in CL for a specific romantic partner. This finding is not consistent with previous research, which revealed gender differences across targets (romantic partner, friends, and strangers/humanity) (Sprecher & Fehr, 2005; Sprecher, Fehr, & Zimmerman, 2007). However, we must keep in mind that past research has been conducted among college students (Neto, 2012b), in contrast to the present research that covers the adult life span. Future investigation is needed to clarify whether gender differences in CL are specific to young people.

As expected, religion influenced positively CL, and believers experienced greater CL than non-believers. This finding is in accord with other studies. For instance, Oman, Thoresen, and Hedberg (2010) show that spiritual meditation encourages greater CL among health professionals, which in turn improves caregiving efficacy. This line of enquiry deserves attention due to its potentially important practical applications.

The study reported here also explored the relationship between CL and being in love, and it was expected that individuals in love would experience higher CL for a romantic partner than those who are not in love. The results confirm this expectation, which suggests that love status affects one’s views and that lovers do wear rose-colored glasses (Hendrick & Hendrick, 1988).

Regarding CL and love styles, findings indicate that Eros was positively associated with CL. For Hendrick and Hendrick (1992), the erotic lover values a strong focus on the partner, and along similar lines Underwood (2009) argues that CL is not the same as “the often hormonally driven” romantic love. However, for Berscheid (2010), both kinds of love can be experienced in intimate relationships. Current findings are in accordance with Berscheid, and suggest that these two kinds of love overlap across the adult life span.

The agapic love style is defined as “an ethereal, altruistic love that takes no thought of the self but only of the beloved other” (Hendrick & Hendrick, 1987, p. 144). Altruism is an important characteristic for both Agape and CL, and in both constructs the partner’s well-being is more important than one’s own well-being. Agapic love has much in common with CL, raising the question of whether they are different constructs. Results reported here show that, although measures of the two constructs were associated, the associations were not so large as to suggest that they are redundant. Furthermore, the multiple regression analyses showed that four love styles (Agape, Pragma, Ludus, and Eros) emerged as significant predictors of CL, and not just of the agapic love style, and thus support the claim that CL is not synonymous with Agape. These results indicate that CL is related to, but distinct from, Agape.

Ludus is a manipulative, game-playing love and the ludic lovers are frequently reluctant “to commit themselves to love” (Lee, 1988, p. 50). Therefore, it was expected that a game-playing approach to love should be negatively correlated with CL. This prediction was confirmed. Similarly, CL was negatively related to pragma. The “shopping-list lover” has a pragmatic approach to love and selects a partner according to required attributes (Hendrick & Hendrick, 1992, p. 66). In this study, CL scores were not related to Mania and to Storge and therefore, the pattern of correlations previously found among college students (Neto, 2012b) seem to be generalizable across the adult life span. In sum, different love styles such as Eros, Ludus, Pragma and Agape were found to be significantly correlated with CL. This is not surprising given that different kinds of love are likely to co-occur in relationships (Berscheid, 2010).

As previously thought, subjective well-being was significantly associated with CL. Past research shows that the experience of CL for others has positive benefits for the self (Sprecher & Fehr, 2006), and the current findings confirm the positive benefits for the subjective well-being across the adult life span. The more CL individuals experienced for a romantic partner, the higher their levels of global, sex and love satisfaction. The emotional components of subjective well-being (positive affect and negative affect) were also correlated with CL, and positive affect was the strongest predictor of CL. Hence, experiencing subjective well-being can be beneficial to CL for a romantic partner.

In sum, this study evidenced more similarities than differences between older partners and young partners regarding CL. First, no differences were found for CL across the adult life span. Second, generally the constellations of correlations between CL and love styles, and subjective well-being were similar across the adult life span. Only two differences were found: Among older partners no associations were found between CL and pragma, and satisfaction with sex life. These findings may be interpreted as reflecting the pragmatism of older lovers (Neto, 2001), who may be slightly less satisfied with sex life (Sprecher & Cate, 2004) than their younger counterparts.

This study makes a contribution to the literature on CL, however, limitations should be acknowledged. First, the Compassionate Love Scale is self-reported and the use of multiple methods would help to enhance our understanding of CL. Second, data were cross-sectional and causal implications cannot be drawn. In particular, we examined whether subjective well-being predicts CL. However, the relationships between subjective well-being and CL are likely to be involved in reciprocal causal relationships. Future longitudinal or experimental works could analyze more causal explanations. Third, the present study examined only CL for a romantic partner. Future research should investigate CL for close others and humanity in general.
